# Enhancement of guinea pig cytomegalovirus infection by two endogenously expressed components of the pentameric glycoprotein complex in epithelial cells

**DOI:** 10.1038/s41598-020-65545-5

**Published:** 2020-05-22

**Authors:** Misaki Okumura, Miku Matsuura-Miura, Reina Makino, Takuya Miura, Kazuma Noguchi, Ryuichi Majima, Tetsuo Koshizuka, Naoki Inoue

**Affiliations:** 1 Microbiology and Immunology, Gifu Pharmaceutical University, Gifu, Japan; 2 Present Address: Akashi City Hall, Hyogo, Japan; 3 Present Address: JCR Pharmaceuticals Co., Ltd., Hyogo, Japan

**Keywords:** Microbiology, Molecular biology

## Abstract

A better understanding of the mechanisms underlying cell tropisms and the efficiency of viral infection is critical for the development of vaccines and antiviral drugs for viral diseases. In this study, we worked on the entry mechanisms of guinea pig cytomegalovirus and found that endogenous expression of a combination of two components (GP131 and GP133) of the pentameric glycoprotein complex, which is required for non-fibroblast cell tropisms, enhanced viral infection more than 10-fold. In addition, D138A alteration in GP131 increased this enhancement by an additional 10-fold. Although differences in the efficiency of viral infection among various cell types are usually explained by differences in viral entry or traffic processes, our experimental evidences dismissed such possibilities. Instead, our findings that i) endogenous expression of GP131 and GP133 after nuclear delivery of viral DNA still enhanced infection and ii) an HDAC inhibitor overcame the need of the endogenous expression led us to hypothesize a novel mechanism that controls the efficiency of viral infection through the activation of gene expression from viral DNA delivered to the nuclei. Further studies of this unexpected phenomena warrant to understand novel but also general mechanisms for cell tropisms of viral infection and determinants that control infection efficiency.

## Introduction

Congenital human CMV (HCMV) infection occurs in 0.2–1% of all births and causes birth defects and developmental abnormalities, making the development of CMV vaccines an important issue. HCMV UL128, UL130, and UL131A are essential for viral entry into endothelial and epithelial cells, as well as for viral transmission to leukocytes^1–5^. They form a pentameric complex (Pentamer) with glycoprotein H (gH) and gL^6^. As the immunization of animals with purified or vectored Pentamer induces a significantly high level of neutralizing antibodies, and as sera from seropositive individuals contain strong neutralizing antibodies against Pentamer, Pentamer-based vaccines have received much attention^7–11^.

In contrast to murine and rat CMVs, guinea pig CMV (GPCMV) causes infection *in utero*, which makes GPCMV animal models useful for studies on congenital CMV diseases^12,13^. Previously we noticed that the GPCMV stock purchased from ATCC was a mixture of two strains, one containing and the other lacking a 1.6 kb locus that encodes GP129, GP131, and GP133, homologs of HCMV UL128, UL130, and UL131A, respectively. Importantly, the locus was required for efficient viral growth in animals but not in fibroblast cell cultures^14,15^. GP129/GP131/GP133 form a pentameric complex with gH/gL^16^, and each of GP129/ GP131/GP133 is required for the infection of macrophages^17^ and epithelial cells^18,19^. We prepared several mutants with a charged amino acid-to-alanine alteration in GP131 and found some differences in the effects of the mutations on the infection of the two cell types, epithelial cells and macrophages, suggesting the existence of cell type-dependent Pentamer recognition or function in GPCMV infection^19^.

To clarify the precise requirements of Pentamer for GPCMV infection and to identify cellular receptors for Pentamer-dependent infection, we examined whether endogenously expressed Pentamer components inhibit GPCMV infection due to receptor binding competition (so-called “interference”) as shown in HCMV Pentamer^20^. During such experiments, we found an unexpected phenomenon in which, instead of interference, co-expression of GP131 and GP133, two components of GPCMV Pentamer, enhanced GPCMV infection of epithelial cells more than 10-fold. As there were no precedents to such a phenomenon, we analyzed its mechanisms to understand how the efficiency of viral infection is controlled.

## Results

### Enhancement of infection in epithelial cells expressing GP131 and GP133

**Figure 1:**
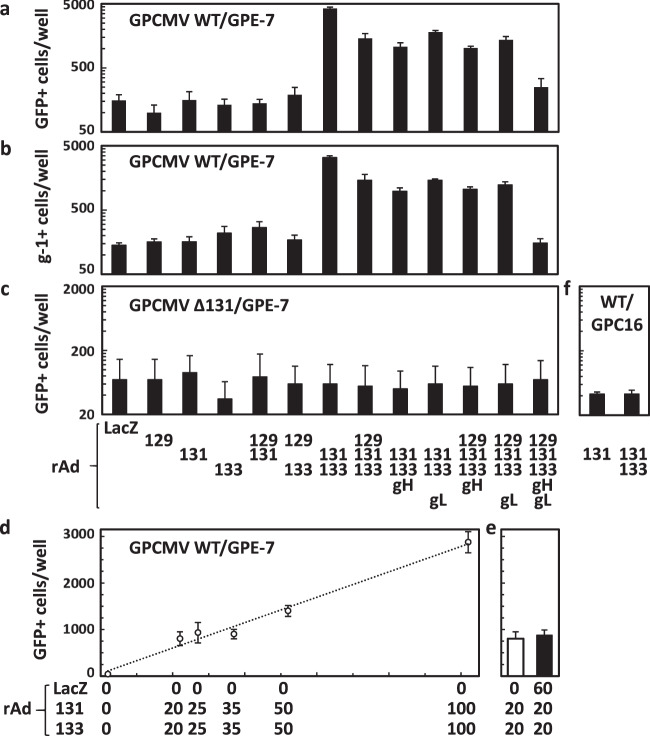
Enhancement of GPCMV infection in epithelial cells expressing GP131 and GP133. (**a**–**c**) GPE-7 cells were plated at 2.0 × 10^4^ cells/well in 96-well plates. Next day, the cells were transduced with rAd-LacZ or with the indicated combinations of rAds encoding a Pentamer component (gH, gL, GP129, GP131, and GP133) at a total MOI of 100. Twenty-four hrs after rAd transduction, the cells were infected with GFP-expressing GPCMV WT (**a,b**) or Δ131 (**c**) at an MOI of 3 (based on titers determined in GPL cells). Two hrs later, the inoculum was replaced with fresh media, and the cells were incubated for additional 48 hrs. The numbers of GFP-positive cells were counted by taking digital images (**a,c**). The cells were also immunostained with monoclonal antibody g-1, which reacts with the GPCMV early antigen, and numbers of g-1-positive cells were counted (**b**). Means and standard deviations (SDs) of numbers in triplicate wells for each condition are shown. **(d,e)** Dose response of GP131/GP133-mediated enhancement. GPE-7 cells were plated and transduced with combinations of rAds encoding LacZ, GP131, and GP133 at the indicated MOIs. Twenty-four hrs later, the cells were infected with GPCMV WT and incubated for 48 hrs. The numbers of GFP-positive cells were counted, as described for (**a**). Means and SDs of numbers of GFP-positive cells in triplicate wells are shown. (**f**) GPC-16 cells were transduced with rAd-131 or with a combination of rAd-131 and rAd-133, infected with GPCMV WT, and incubated as described for GPE-7 cells in Panel a. Means and SDs of numbers of GFP-positive cells in triplicate wells for each condition are shown.

Guinea pig epithelial cells (GPE-7) were transduced with a recombinant adenovirus (rAd) expressing a component of GPCMV Pentamer or with various combinations of these rAds, and then infected with GFP-expressing GPCMV, GPCMV-BACd9kWT (GPCMV WT)^17^, to examine the effects of endogenous expression of the Pentamer components on the efficiency of GPCMV infection. In the cells transduced with any combination containing both the rAds expressing GP131(rAd-131) and GP133 (rAd-133), but not in those transduced with rAd-131 or rAd-133 individually, GPCMV infection resulted in a 6- to 30-fold increase in the number of GFP-positive cells in comparison with those in the cells transduced with the rAd expressing β-galactosidase (rAd-LacZ) (Fig. 1a). The number of GFP-positive cells in GPE-7 cells without rAd transduction was the same as those in rAd-LacZ-transduced cells (data not shown). Expression of all five Pentamer components did not interfere with the GPCMV infection, rather the infection was enhanced slightly. These results based on GFP expression were confirmed by immunostaining of the infected cells with g-1, a monoclonal antibody against a viral early antigen^21^ (Fig. 1b). There was an almost complete correlation between the numbers of GFP-positive cells and those of g-1 (viral early antigen)-positive cells (correlation coefficient r = 0.99), indicating that the GFP-positive cell number can represent the number of GPCMV-infected cells.

To clarify whether the enhanced infection occurred in a Pentamer-dependent manner, the same experiment as that of Fig. 1a except for infection with GPCMV-BACd9k GP131Stop (GPCMV Δ131), a GPCMV mutant lacking GP131 expression due to the introduction of an early stop-codon and a frameshift, was conducted. Previously, we demonstrated that GPCMV Δ131 infected GPE-7 cells at 20–30% of the efficiency of infection with GPCMV WT^19^. As shown in Fig. 1c, the efficiency of infection with the GP131 mutant virus was not enhanced by the co-expression of GP131 and GP133, suggesting that GP131/GP133-mediated enhancement cannot compensate for the lack of the Pentamer component in the virions. In contrast, infection with TurboRFP-expressing GPCMV, which was constructed by homologous recombination, confirmed to encode the proper Pentamer genes, and can infect macrophages and epithelial cells^13^, was also enhanced in the GPE-7 cells transduced with rAd-131 and rAd-133 (data not shown). Experiments to examine the effects of the multiplicity of infection (MOI) of rAds demonstrated that the GP131/GP133-mediated enhancement of infection was dose-dependent (Fig. 1d) and that the adjustment of the total MOIs of rAds with rAd-LacZ had no effect on the enhancement of infection (Fig. 1e).

In the only commercially available guinea pig epithelial cell line GPC-16 in which GPCMV infection was not Pentamer-dependent^17^, the GP131/GP133-mediated enhancement was not observed (Fig. 1f).

### The enhancement of infection is not due to increased endocytosis

**Figure 2:**
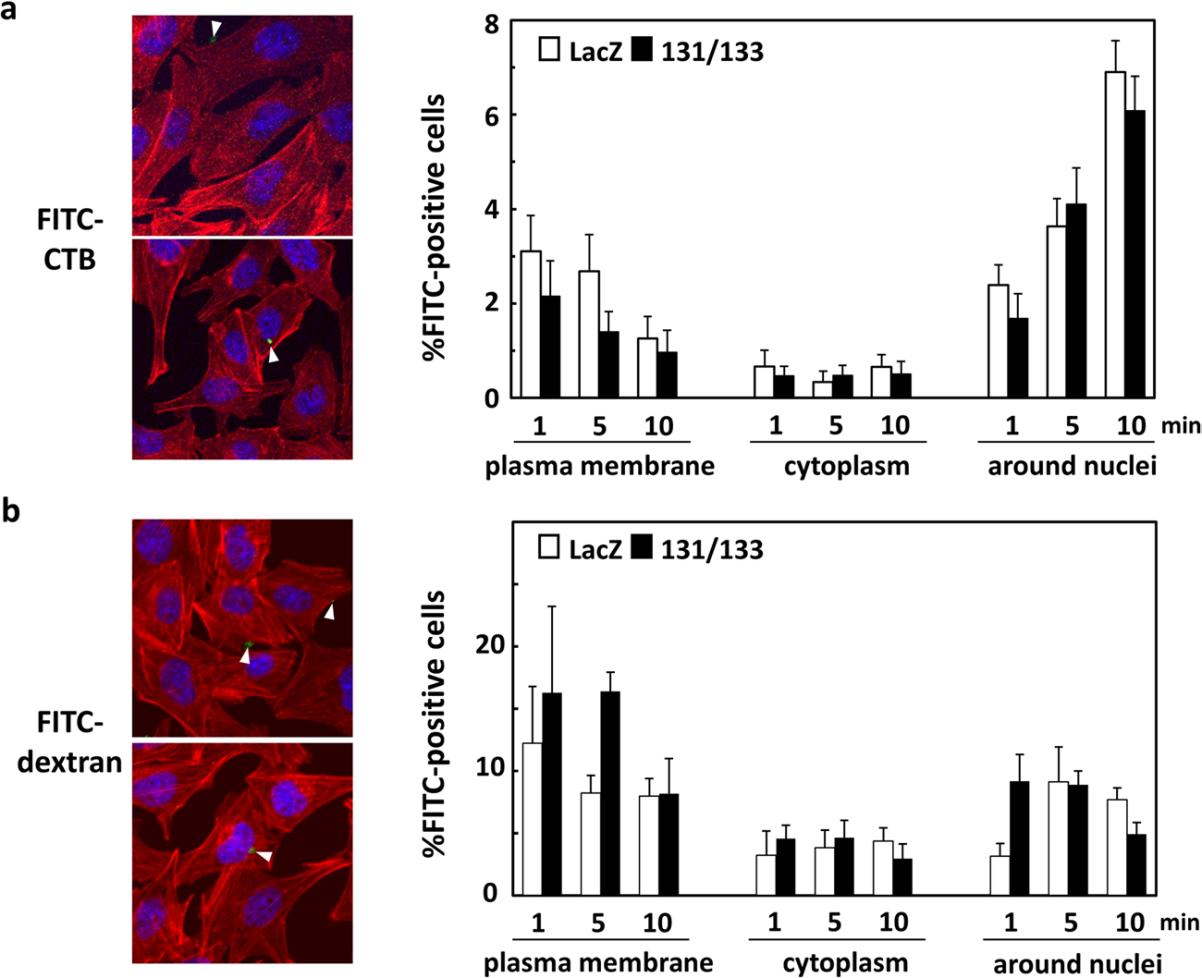
No enhancement of caveolae- and macropinocytosis-dependent endocytosis pathways. (**a**) GPE-7 cells were plated in 8-well chamber slides and, 24 hrs later, transduced with rAd-LacZ or with a combination of rAd-131 and rAd-133 at a total MOI of 100. At 24 hrs after rAd transduction, the culture media of the cells were replaced with pre-warmed FBS-free DMEM/F12 (1:1) medium containing FITC-CTB. At the indicated minutes after the addition of the FITC-conjugated substance, the culture media of the cells were removed, and the cells were fixed with 3.7% formalin in PBS for 15 min, rinsed with PBS containing 3% BSA twice, and treated with 0.5% Triton X-100 in PBS for 15 min. After PBS washing, the cells were reacted with acti-stain TM555 phalloidin (red) and DAPI (blue) and observed under a confocal microscope. Fifteen images were randomly captured for each condition, and the total numbers of the cells (DAPI-positive) and those containing FITC-conjugated materials on the plasma membrane, in the cytoplasm, and around the nuclei were counted. Examples of images of the cells containing FITC-conjugated materials, which are indicated with arrowheads, on the plasma membrane and around the nuclei, and means and standard errors of mean (SEMs) of the percentages of the FITC-positive cells in the 15 images for each condition are shown. Each image contained around 50 cells, and around 45 FITC signals detected in a total of around 750 cells for each condition were classified. **(b**) The same experiment as (**a**) except for the use of FITC-70kDa dextran in place of FITC-CTB was performed.

To examine whether the co-expression of GP131 and GP133 increases endocytotic pathway activity in the absence of infection, FITC-conjugated Cholera toxin B subunit (CTB) or 70-kDa dextran was applied to the GP131/GP133-expressing cells or to the LacZ-expressing cells, and the cellular traffic of the FITC-conjugated substances from the plasma membrane to near the nuclei in the cells was monitored. Picture images show examples of the traffic of these FITC-conjugated substances. There were no significant differences in the traffic speed of FITC-CTB (Fig. 2a) or FITC-dextran (Fig. 2b) between the two conditions; *i.e*., in rAd-LacZ- and rAd-131/rAd-133-transduced cells, suggesting that GP131/GP133 co-expression has no effect on caveolae-dependent endocytosis or macropinocytosis.

### Centrifugation immediately after infection additively improves infectivity

**Figure 3:**
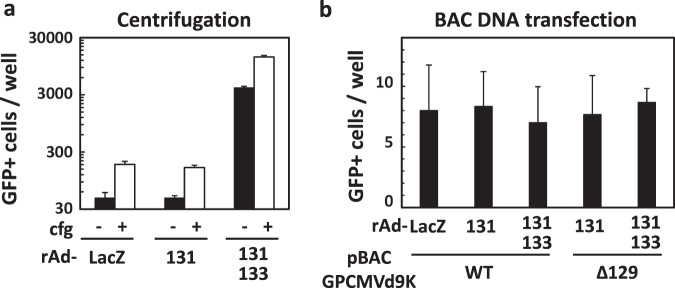
Effects of low-speed centrifugation and transfection of viral genome DNA on enhancement of infection. GPE-7 cells were plated in 96-well plates and, next day, transduced with rAd-LacZ, rAd-131, or a combination of rAd-131 and rAd-133 at a total MOI of 100. **(a)** Twenty-four hrs later, the cells were infected with GPCMV WT at an MOI of 3, incubated (cfg-) or centrifuged at 400 × *g* (cfg+) for 30 min at room temperature, and then incubated at 37 °C for 90 min. The inoculum was replaced with fresh media, and then incubated at 37 °C for 48 hrs. Means and SDs of numbers of GFP-positive cells counted in triplicate wells are shown. **(b)** Twenty-four hrs after rAd transduction, the cells were transfected with 0.1 μg of pBAC-GPCMVd9K WT (WT) or -GPCMVd9K GP129Stop (Δ129) DNA using FuGene 6 (Promega). Five days later, the numbers of GFP-positive cells were counted. Means and SDs of numbers of GFP-positive cells in 6 wells for each condition are shown.

It is well known that slow centrifugation immediately after infection enhances CMV infectivity, although the mechanism for this remains unclear^22^. As shown in Fig. 3a, a 3- to 5-fold increase in infectivity was observed by 30 min-centrifugation immediately after infection in the cells transduced with rAd-LacZ, rAd-131, or a combination of rAd-131 and rAd-133, suggesting that the mechanism of the GP131/GP133-mediated enhancement differs from that of the centrifugation-mediated enhancement.

### The enhancement depends on infection with virions but not with viral DNA

To investigate whether the GP131/GP133-mediated enhancement of infection can also be initiated by the delivery of viral DNA instead of virions, BAC DNA containing the GPCMV WT genome (WT) was transfected into the cells transduced with rAd-LacZ, rAd-131, or a combination of rAd-131 and rAd-133. However, no significant differences in the numbers of the GFP-positive cells were observed (Fig. 3b). In addition, transfection of pBAC-GPCMV lacking GP129 expression (Δ129) resulted in no significant differences in the numbers of the GFP-positive cells between the cells transduced with rAd-131 and those with a combination of rAd-131 and rAd-133 (Fig. 3b). Combined with the fact that the GP131 gene product is incorporated in the virions^19^ and the requirement for the GP131 gene in the GPCMV genome (Fig. 1c), these results led us to speculate that the engagement of cellular receptors with virions is required for the GP131/GP133-mediated enhancement of infection.

### The enhancement is not due to an increase in viral attachment

**Figure 4:**
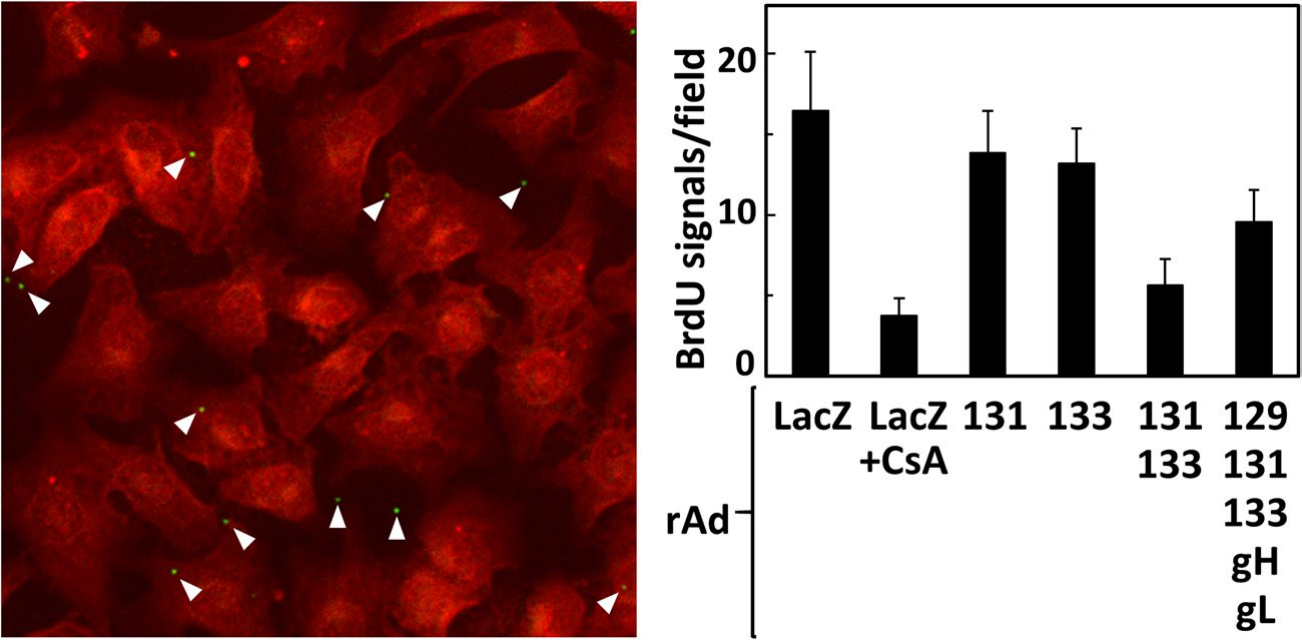
The GP131/GP133-mediated enhancement of infection is not due to an increase in attachment. GPE-7 cells in 8-well chamber slides were transduced with combinations of the indicated rAds. Twenty-four hrs later, the cells were infected at 4 °C with BrdU-labeled GPCMV WT at an MOI of 0.01, incubated at 4 °C for 2 hrs in the absence or presence of 1 mg/ml chondroitin sulfate A (CsA), then rinsed with cold PBS, and finally fixed with acetone. Detection of BrdU-labeled GPCMV was performed as described in Materials & Methods. Images of 20 fields (0.17 μm^2^/field) for each condition were captured. An example of the image as well as means and SDs of numbers of the BrdU signals counted for each condition are shown.

The efficiencies of viral attachment to the GPE-7 cells expressing various combinations of the Pentamer components were compared. For this purpose, bromodeoxyuridine (BrdU)-labeled GPCMV WT stocks were prepared. Chondroitin sulfate A (CsA), an attachment inhibitor, was added to demonstrate the assay specificity. Although the average numbers of BrdU-labeled GPCMV particles attached to the cells transduced with rAd-LacZ, rAd-131, or rAd-133 were similar, those attached to the cells transduced with the combinations containing both rAd-131 and rAd-133 were reduced rather than increased (Fig. 4). Although the reduction was statistically significant (one way ANOVA, *p* < 0.001), we did not pursue the mechanism behind this observation.

### The enhancement remains dependent on endocytosis

**Figure 5:**
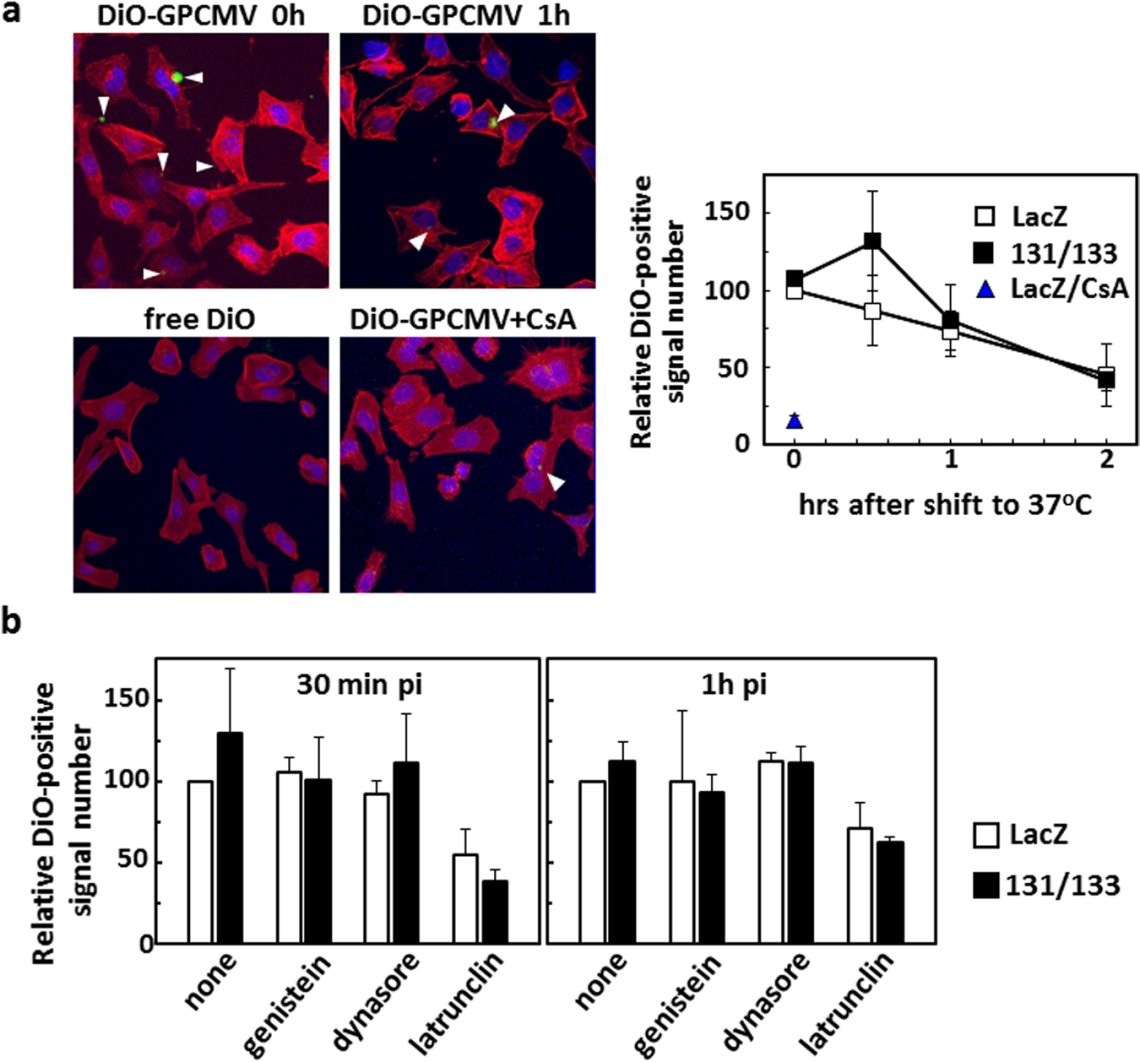
No effects of GP131/GP133 expression on viral cytoplasmic traffic. (**a**) GPE-7 cells were transduced with rAd-LacZ or a combination of rAd-131 and rAd-133. One day later, the cells were incubated with DiO-labeled GPCMV WT at an MOI of 22.5 on ice for 1 hr in the absence or presence of CsA. After replacement of the inocula with pre-warmed medium, the culture temperature was shifted up to 37 °C to allow virion traffic. The cells were fixed at the indicated time points and stained with DAPI and fluorescent phalloidin. Images of 10 fields (0.17 μm^2^/field) for each condition were captured (examples are shown), and means and SDs of relative DiO-positive signal numbers in two independent experiments are plotted using the DiO-positive signal number in the cells transduced with rAd-LacZ at time 0 hr as a 100% control. Most DiO-positive cells contained one signal per cell. “time 0 hr” indicates the time point at the temperature shift-up. **(b)** One day after transduction of GPE cells with rAds as described for (**a**), their culture media were replaced with pre-warmed medium containing the indicated inhibitors. The cells were incubated at 37 °C for 30 min and then placed on ice. Next, the cells were incubated with DiO-labeled GPCMV WT at an MOI of 22.5 on ice for 1 hr. After replacement of the inocula with pre-warmed medium containing the same inhibitor, the cells were incubated for 30 min or 1 hr, fixed, and treated as described for (**a**). Images were captured as described for (**a**), and means and SDs of relative DiO-positive signal numbers in three independent experiments are shown using the DiO-positive signal number in the cells that were transduced with rAd-LacZ and incubated without any inhibitor (none) as a 100% control.

As no effects were observed on the viral attachment processes for the co-expression of GP131 and GP133, next we analyzed the effects of their co-expression on the viral internalization process; *i.e*., endocytosis, during infection. Intracellular traffic of DiO-labeled GPCMV in the LacZ- and GP131/GP133-expressing cells was monitored. As DiO is a fluorescent probe for membrane, its signal with a certain mass can be detectable until its diffusion, *i.e.* at the point of fusion of viral envelope with cellular membranes, in a confocal microscopic analysis. In other words, we expected that decay of DiO signals represented the traffic speed of virions from entry to membrane fusion. To make sure the specificity of the assay, first, cells were reacted with a free form of DiO of the amount equal to those in the DiO-labeled GPCMV stock used for infection. Any DiO signals were undetectable as dots in confocal microscopic analyses (Fig. 5a). In addition, CsA treatment of the cells significantly reduced the number of DiO signals in the DiO-GPCMV-infected cells to 15% of those without the treatment (Fig. 5a, time 0 hr). Both observations suggest that DiO signals observed are GPCMV-specific. At any time points during the first 2 hrs after shift-up to 37^o^C, there were no significant differences in the traffic speed of GPCMV virions in the cells irrespective of GP131/GP133 expression (Fig. 5a). The effects of co-expression of GP131 and GP133 on endocytosis were examined by monitoring the presence of DiO-labeled GPCMV inside the cells after infection in the presence of genistein, dynasore, or latrunculin A (Fig. 5b). A broad-spectrum tyrosine kinase inhibitor genistein and a dynamin GTPase inhibitor dynasore interfere with caveolae- and clathrin-mediated endocytosis, respectively. In contrast, an actin polymerization inhibitor latrunculin A interferes with many other endocytotic mechanisms, including macropinocytosis^23^. Irrespective of the co-expression, latrunculin A decreased the DiO signals (Welch’s t-test, *p* < 0.05), while genistein and dynasore had no significant effects. These observations suggest that GPCMV infection is dependent on a caveolae- and clathrin-independent endocytotic mechanism. However, there was no difference in the effect of latrunculin A between the cells transduced with rAd-LacZ and those with a combination of rAd-131 and rAd-133. These findings suggest that the GP131/GP133 co-expression did not change the nature of the endocytosis-dependent GPCMV infection.

### Amino acid sequences of GP131 required for the enhancement of infection

**Figure 6:**
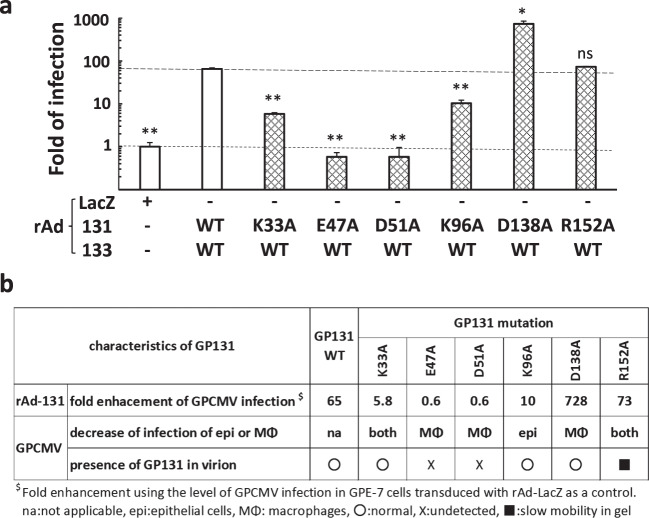
GP131 amino acid sequences required for the enhancement. (**a**) GPE-7 cells were plated at 2.0 × 10^4^ cells/well in 96-well plates. Next day, the cells were transduced with rAds-LacZ or with the indicated combinations of rAd encoding the indicated GP131 (WT or mutant with K33A, E47A, D51A, K96A, D138A, or R152A) and rAd-133 at a total MOI of 100. Twenty-four hrs after rAd transduction, the cells were infected with GPCMV WT at an MOI of 3. Two hrs later, the inoculum was replaced with fresh medium, and the cells were incubated for 48 hrs. Means and SDs of numbers of GFP-positive cells counted in triplicate wells for each condition are shown. Data were analyzed by Welch’s t-test. *p < 0.01, **p < 0.005, ns: not significant. **(b)** Fold of the enhancement of GPCMV infection in the cells transduced with a combination of rAd expressing mutated GP131 and rAd-133 were compared with our previous findings on the effects of GP131 mutations on infection of epithelial cells and of macrophages^19^. The fold of enhancement with the GP131 mutants is shown using that of the LacZ as a control.

rAds expressing GP131 with a charged amino acid-to-alanine alteration prepared previously^19^ were used to identify the amino acid sequences required for the enhancement of infection. The rAd-mediated expression levels of all mutated GP131 proteins in human epithelial cell line HEK-293A were similar, while that of R152A was slightly less than those of the others and the mobilities of E47A and D51A in SDS-PAGE were slower than those of the wild-type (WT) and other mutated versions^19^. As shown in Fig. 6a, the E47A and D51A alterations abolished, and the K33A and K96A alterations reduced the enhancement of infection. Interestingly, the D138A alteration enhanced the GPCMV infection by an additional 10-fold. There were no apparent correlations between the enhancement and the requirements for infection that we reported previously^19^ (Fig. 6b). As the amounts of GP131 WT and mutated versions were similar in HEK-293A cells transduced with their rAds^19^, we assume that the differences in the folds of enhancement were not due to different amounts of GP131 in cells. As the amounts of GP131 and other mutated versions in GPE-7 cells transduced with their rAds were marginal to be detected in immunoblotting and immunofluorescent assays, we could not compare them in GPE-7 cells.

### Similar time course of appearance of GFP and viral antigens irrespective of the enhancement

**Figure 7:**
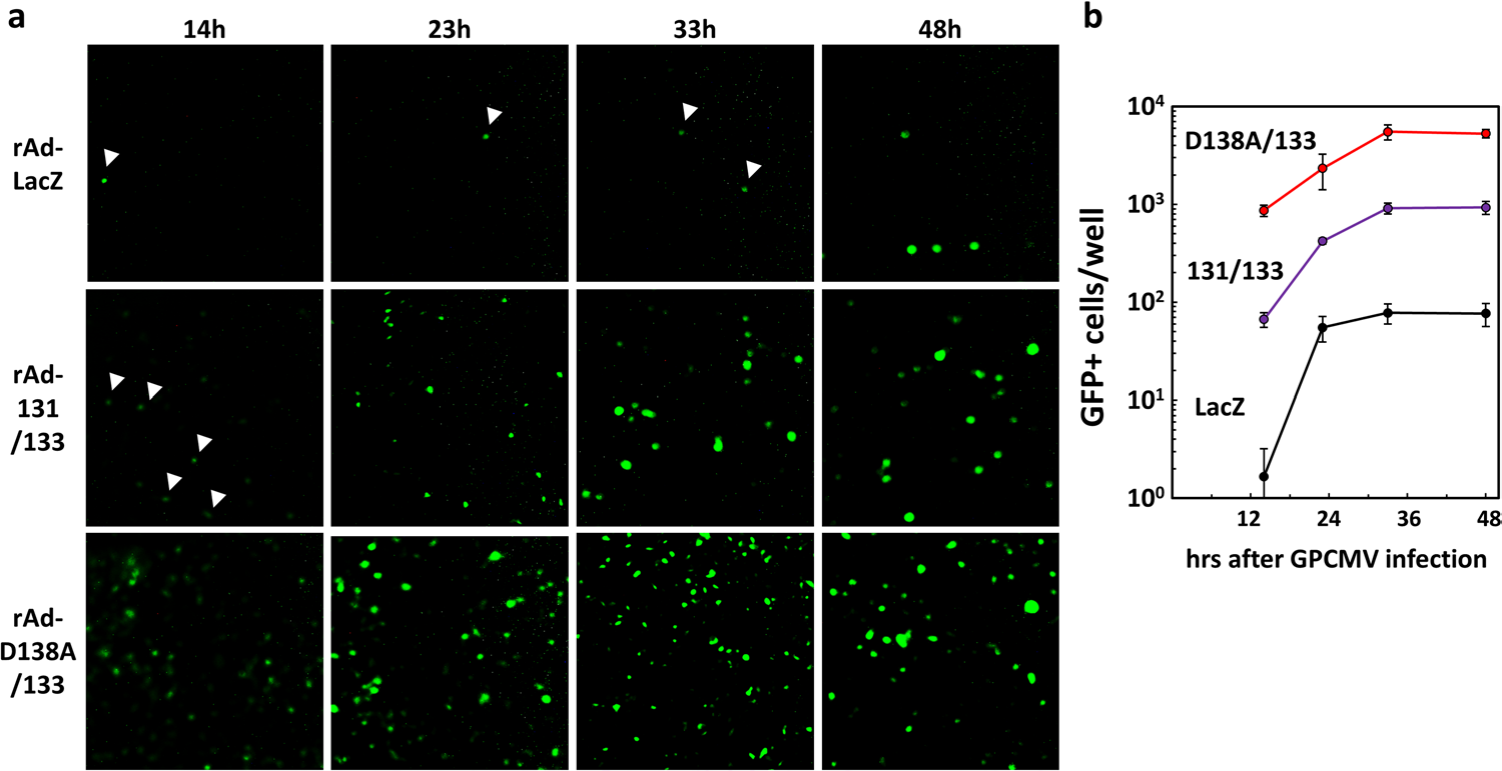
Time course of GFP expression. (**a**) GPE-7 cells were plated in 96-well plates and transduced with rAd-LacZ, a combination of rAd-131 and rAd-133, or a combination of rAd-D138A and rAd-133 at a total MOI of 100. Twenty-four hrs later, the cells were infected with GPCMV WT at an MOI of 3. At the indicated time points after GPCMV infection, images of GFP expression in the infected cells were captured. Arrowheads indicate cells weakly expressing GFP. **(b)** Means and SDs of numbers of GFP-positive cells counted in triplicate wells are shown.

As there were no differences in viral attachment or internalization to the cytoplasm, we next considered two possible scenarios. One potential scenario was that the nuclear delivery of viral DNA occurs more rapidly in the GP131/GP133-expressing cells. This scenario can expect time-dependent differences in the expression of GFP and viral antigens between the cells expressing LacZ and those expressing GP131/GP133. The other scenario was that expression of the delivered viral DNA is more preferentially activated in the GP131/GP133-expressing cells than in the control cells. To distinguish between these two possibilities, the time course of the appearance of GFP-positive cells after GPCMV WT infection was compared among the cells transduced with rAd-LacZ, a combination of rAd-131 and rAd-133, and a combination of rAd expressing GP131 D138A (rAd-D138A) and rAd-133 (Fig. 7). At 9~10 hrs after GPCMV WT infection, irrespective of GP131/GP133 expression, GFP-expression was almost undetectable under a microscope by the naked eye and only faintly detectable with the aid of a sensitive camera, but the number of GFP-positive cells was difficult to count. At 14 hrs post-infection, GFP-positive cells were detectable, but with some difficulty, while at 23 hrs post-infection, they were readily detectable. Thereafter, at 33 and 48 hrs post-infection, there was little increase in the number of GFP-positive cells. Although the number of GFP-positive cells differed among the cells transduced with the three combinations of rAds, the time course of the appearance and saturation of GFP-positive cells was similar for each, suggesting that the enhancement of infection is not due to time-dependent differences.

**Figure 8:**
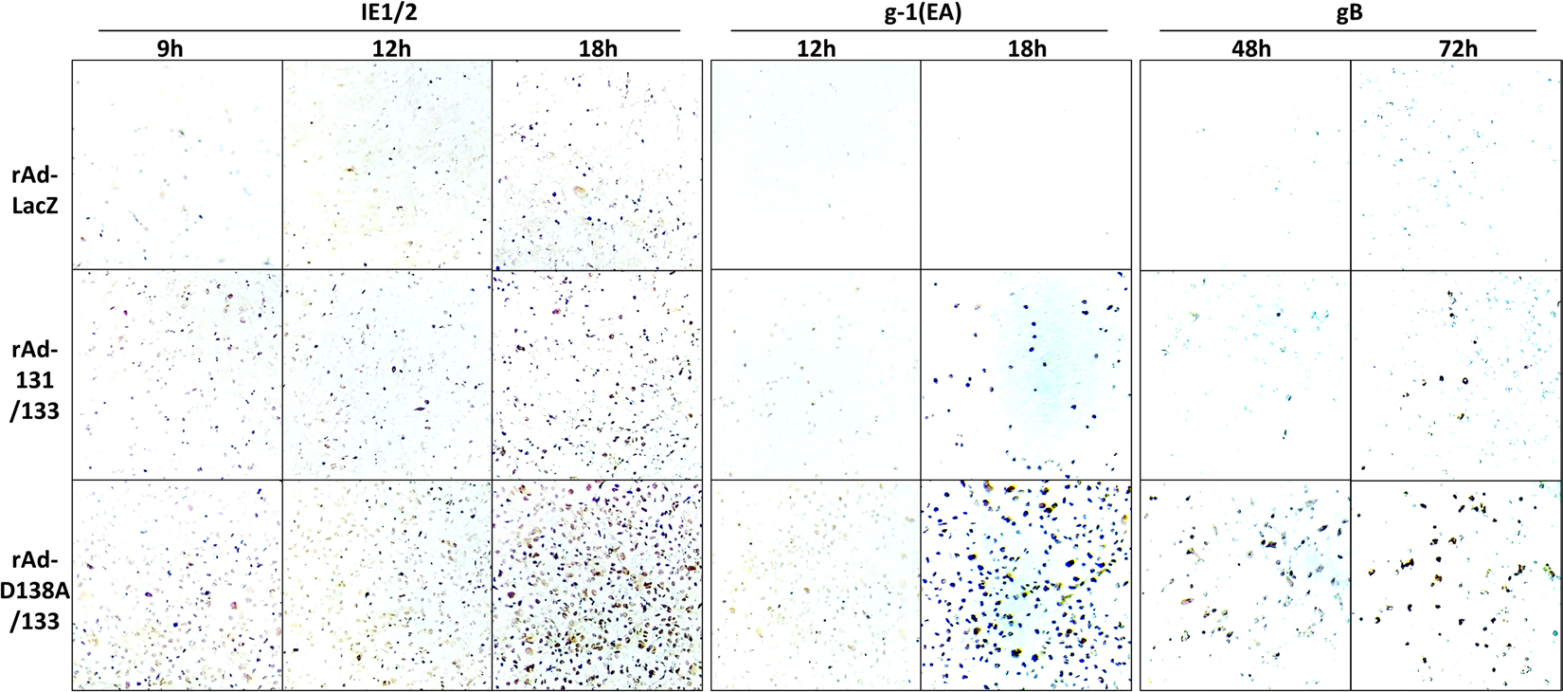
Time course of viral antigen expression. GPE-7 cells were plated in 24-well plates and transduced with rAd-LacZ, a combination of rAd-131 and rAd-133, or a combination of rAd-D138A and rAd-133 at a total MOI of 100. Twenty-four hrs later, the cells were infected with GPCMV WT at an MOI of 3. At the indicated time points post-infection, the cells were fixed and immunostained with anti-IE1/2 polyclonal antibodies, with g-1 (anti-early antigen) monoclonal antibody, or with anti-gB polyclonal antibodies.

As CMV undergoes an ordered cascade of expressions of viral antigens, *i.e*. immediate early (IE), early (EA), and late (LA) gene antigens, upon infection of permissive cells, the appearance of cells expressing IE, EA or LA antigens was also compared among the cells transduced with rAd-LacZ, a combination of rAd-131 and rAd-133, and that of rAd-D138A and rAd-133 at indicated hrs after GPCMV WT infection. IE1/2 antigens were hardly detectable at 9 hrs, evident at 12 hrs, and clearly visible at 18 hrs after GPCMV infection among the cells transduced with the three combinations of rAds, confirming that the time course of GFP expression for each was similar to that of IE expression (Fig. 8). In addition, the EA antigen recognized by monoclonal antibody g-1 became faintly detectable at 12 hrs and clearly visible at 18 hrs post-infection. The early/late antigen gB^24^ was weakly detectable at 48 hrs and strongly at 72 hrs post-infection. The number of cells expressing the EA or early/late antigen was bigger in an order of the cells transduced with rAd-LacZ, a combination of rAd-131 and rAd-133, and that of rAd-D138A and rAd-133, indicating that all phases of viral infection were enhanced by the endogenous co-expression of GP131 and GP133.

**Figure 9:**
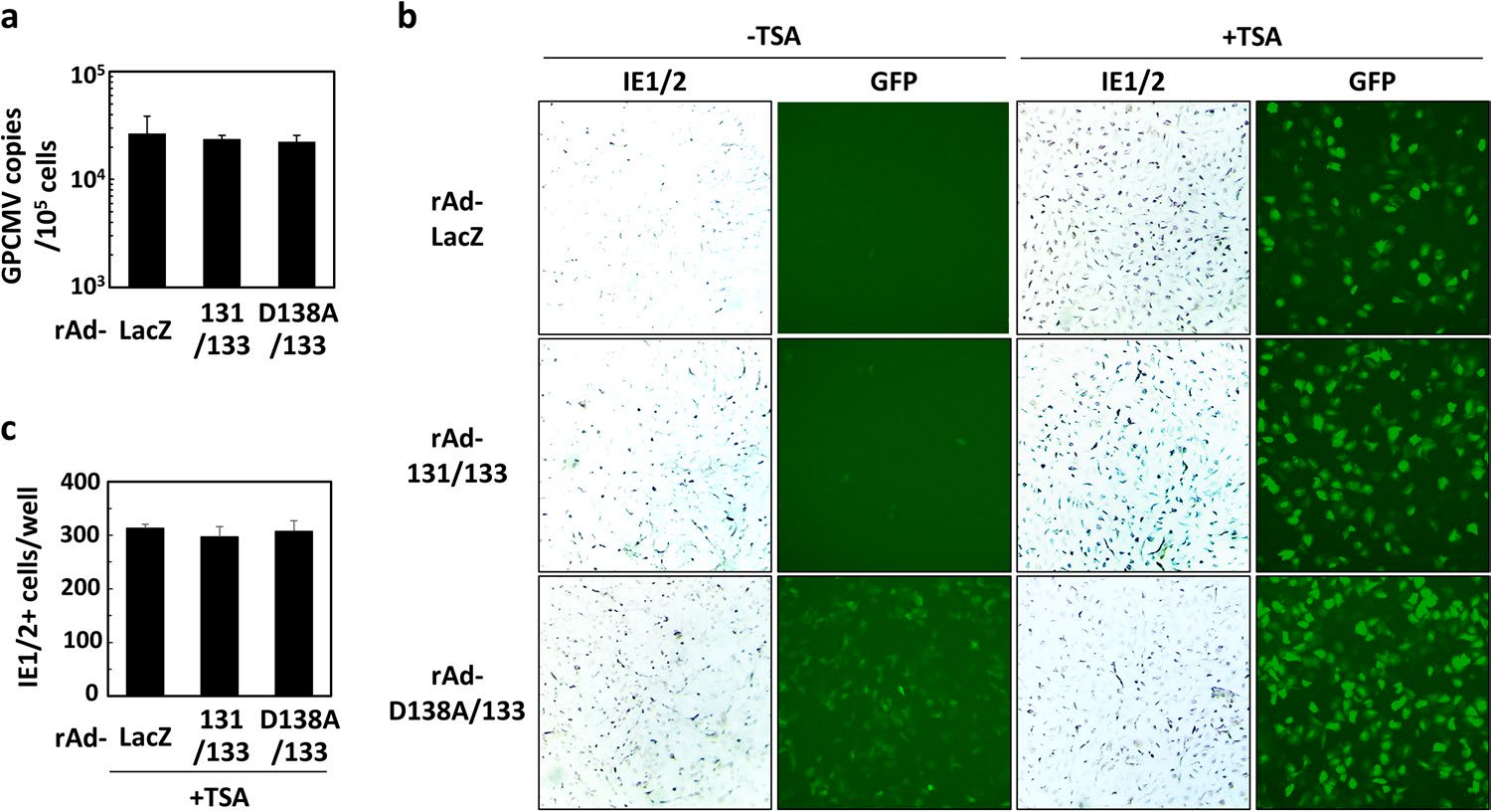
Capabilities of viral gene expression from viral DNA delivered to the nuclei. (**a**) GPE-7 cells were transduced with rAds and infected with GPCMV WT as described in the legend for Fig. 8. At 12 hrs post-infection, the nuclear fractions of the infected cells were collected from triplicate wells, and viral loads were determined as described in the Materials and Method section. Means and SDs of GPCMV copy numbers per 10^5^ cells are shown. **(b,c)** GPE-7 cells were transduced with rAds at a total MOI of 100. Twenty hrs later, TSA was added to the cultures at a final concentration of 0.066 μM. Twelve hrs after the addition, the cells were infected with GPCMV WT at MOIs of 3 or 0.03. At 14 hrs post-infection, images of GFP expression in the infected cells were captured, and the infected cells were fixed and immunostained with anti-IE1/2 polyclonal antibodies. The images of GFP-positive cells and IE1/2-positive cells infected at an MOI of 3 (panel b) and comparison of the number of IE1/2-positive cells infected at an MOI of 0.03 among TSA-treated cells (panel c) are shown.

To see if the differences in the number of GFP- or IE1/2-positive cells can be ascribed to the copy numbers of viral DNA in the nuclei, the average viral copy number in the nuclei per cell at 12 hrs post-infection was compared. There were no differences in viral loads (Fig. 9a), indicating that the endogenous co-expression of GP131 and GP133 has no effects on the viral nuclear delivery. To confirm whether viral genomes delivered to the nuclei have equal capabilities of viral gene expression irrespective of endogenous co-expression of GP131 and GP133, GPE-7 cells transduced with combinations of rAds were treated without or with trichostatin A (TSA), which selectively inhibits the class I and II mammalian histone deacetylase (HDAC) families of enzymes, for 12 hrs, as it is well known that HDAC is involved in repression of the IE1/2 gene promoter in non-permissive cells^25^. After the TSA treatment, the cells were infected with GPCMV WT at MOIs of 3 or 0.03, and the numbers of GFP-positive cells infected with GPCMV WT at an MOI of 3 were compared at 14 hrs post-infection (Fig. 9b). There were no apparent differences in the number of GFP-positive cells among the cells treated with TSA, and that number was significantly bigger than that of the TSA-untreated cells. In addition, the number of GPCMV IE1/2-positive cells was the same among the cells that were transduced with rAds, treated with TSA, and infected with GPCMV WT at an MOI of 0.03 (Fig. 9c), indicating that the viral DNA delivered to the nuclei has equal potential capabilities of viral gene expression.

### Late expression of GP131/GP133 still enhanced infection

**Figure 10:**
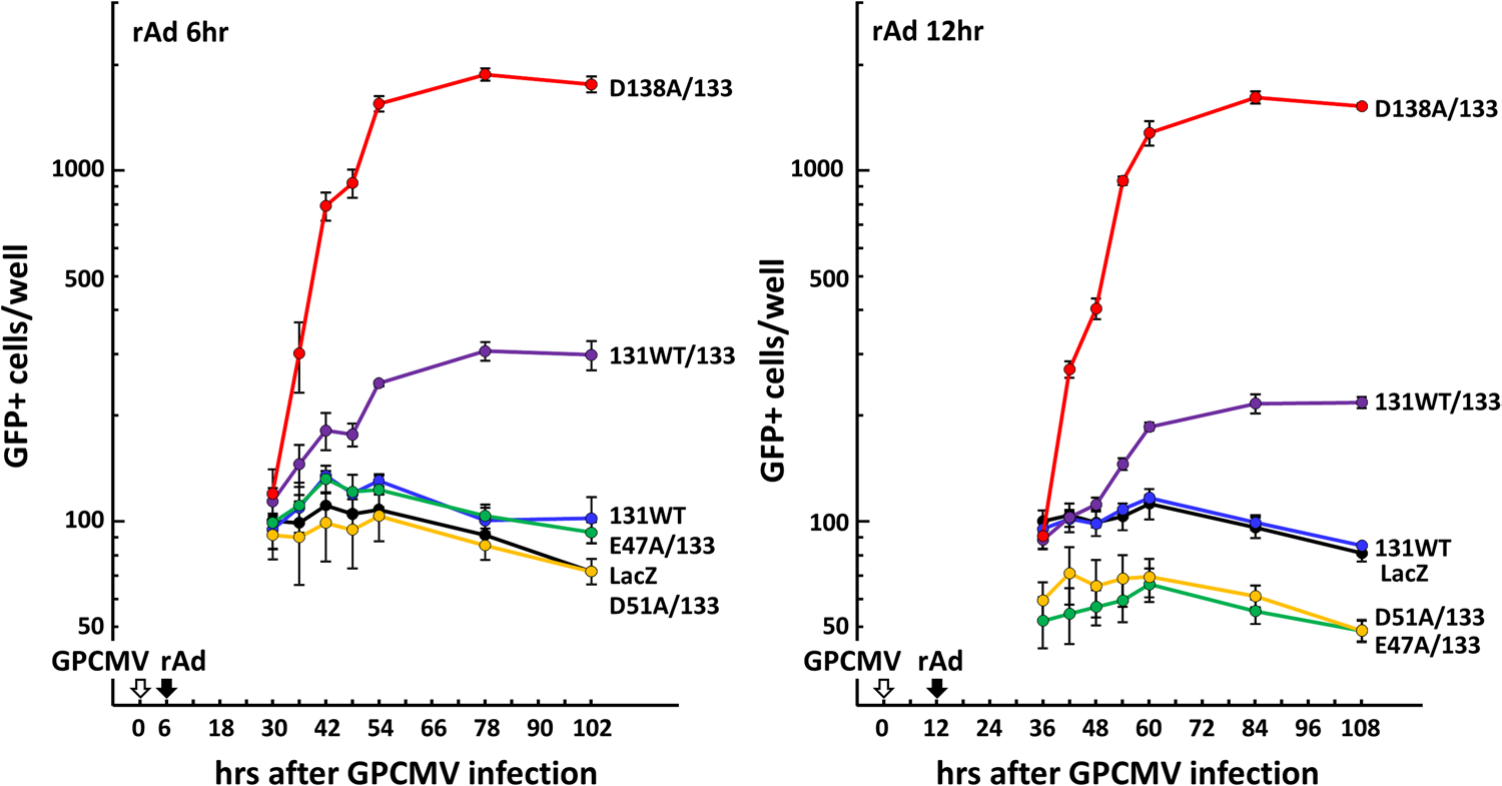
Co-expression of GP131 and GP133 after viral nuclear delivery still enhanced GPCMV infection. GPE-7 cells were plated at 2 × 10^4^ cells/well of 96-well plates and, 24 hr later, infected with GPCMV WT at an MOI of 3 (time point 0 hr), and the inocula were then replaced with fresh medium 2 hrs later. Culture media were removed at 6 hrs or at 12 hrs after GPCMV infection, and the cells were rinsed with PBS and transduced with combinations of the indicated rAds at a total MOI of 100. Relative numbers of GFP-positive cells at the indicated hrs after GPCMV infection are plotted using those of the cells transduced with rAd-LacZ at 24 hrs after rAd transduction as a 100% control. Means and SEMs of relative numbers in triplicate wells are shown.

To see if co-expression of GP131 and GP133 enhances viral gene expression from the viral DNA delivered to the nuclei, GPE-7 cells were transduced with combinations of rAds at 6 hrs and 12 hrs after GPCMV WT infection. As shown in Fig. 8, IE1/2 expression was already evident at 12 hrs after GPCMV infection. If co-expression of GP131 and GP133 increases viral DNA delivery to the nuclei physically, it is expected that their expression after viral DNA delivery may not increase GPCMV infection. However, co-expression of GP131 and GP133 enhanced GPCMV infection, the D138A mutation in GP131 showed additional enhancement, whereas the E47A and D51A mutations both abolished the enhancement (Fig. 10). Importantly, the time courses between the two time points of rAd transduction were similar. These results suggest that viral DNA delivered to the nuclei is more preferentially activated for expression in the GP131/GP133- and GP131 D138A/GP133-expressing cells.

## Discussion

In this study, we found unexpectedly that expression of GP131 and GP133, two of the Pentamer components, enhanced the efficiency of GPCMV infection in epithelial cells. The enhancement was concluded not only based on the expression of GFP gene cloned into GPCMV WT, but also based on the expression of viral IE, EA, and early/late gene products. However, as shown in our previous study^19^, viral growth in GPE-7 cells was very slow, and it took at least 7 days to observe cell-to-cell spread, which makes hard to examine the effects on viral yields in a multiple step growth curve assay. We demonstrated that i) GP131/GP133 expression did not increase viral attachment or cellular virion traffic processes, and ii) endogenous co-expression of GP131 and GP133 did not compensate for the lack of GP131 in the GPCMV used for infection, which is different from trans-complementation of the defect in MCMV m131/m129 encoding Mck-2, a homolog of UL130 and GP131, to promote viral entry into macrophages as a gH/gL/MCK-2 complex^26^. We also demonstrated that the GP131/GP133-enhanced the infection of GPE-7 cells in an endocytosis-dependent manner as in normal infection. Previously, it was reported that bafilomycin treatment dramatically inhibited GPCMV infection of epithelial cells but did not greatly impact on fibroblast infection^18^. More vigorous studies are required to see whether the endocytotic pathway for the GPCMV infection of GPE-7 cells is similar to that for HCMV entry into some cell types^27–29^. Although it has been reported that HCMV is initially retained in early endosomes and then moves sequentially to the trans-Golgi network and recycling endosomes before nuclear translocation in monocytes, and that the Src signaling pathway induced by viral attachment drives a unique nuclear translocation pattern^30,31^, this study was not intended to scrutinize such details. A comparison of the time course of GFP and viral antigen expression suggests that the enhancement of infection is not dependent on early increase of viral gene products expressed from the viral genome. Furthermore, enhancement of GPCMV infection, even by transduction with rAd-131 and rAd-133 at 12 hrs after GPCMV infection; *i.e*., after IE gene expression, supports the notion that the viral DNA delivered to the nuclei is more preferentially activated for viral IE-1/2 gene expression in the GP131/GP133-expressing cells. Also, an HDAC inhibitor overcame the need of the endogenous co-expression of GP131/GP133 for the increase of the numbers of the IE-1/2-expressing cells. Taking account of those findings, we hypothesize that expression of GP131/GP133 induces activation of viral gene expression from viral DNA delivered to the nuclei. However, we cannot exclude an alternative hypothesis that GP131/GP133 transduction influences release of viral genomes from capsids into the nucleus, as it was technically difficult to determine the copy numbers of nuclease-resistant viral genomic DNA. Although in the case of HCMV, it is reported that tumor necrosis factor alpha potentiates phosphorylation of KAP-1, a master co-repressor that silences IE gene expression by recruiting HP-1 and histone methyltransferase to the viral genome, for reactivation of virus from the latent myeloid cells^32,33^, our study found for the first time that endogenous expression of virion components activates GPCMV gene expression that is partly silenced in infected cells. It has been reported that induction of the HCMV signaling pathways for efficient infection depends on Pentamer components^30,34^. As GP131 and GP133 are gene products expressed at the late phase of infection, it is unlikely that their late expression induces such signaling pathways. However, as the precise roles of UL130 and UL131A, the HCMV homologs of GP131 and GP133, in the entry and signaling processes remain unclear, it is still possible to hypothesize that expression of GP131/GP133 induces the signal pathways for activation of viral gene expression that is achieved by the engagement of Pentamer with its receptors or by interactions with cellular molecules for signaling. Alternatively, endoplasmic reticulum stress induced by endogenous expression of GP131/GP133 may activate viral gene expression through the unfolded protein response (UPR), as it has been documented that HCMV and MCMV infection, or their particular gene products, activates UPR and modulates viral gene expression^35,36^. However, such an interpretation could be inconsistent with our observation that the amounts of GP131 and its mutated versions with a single amino acid alteration were similar in the HEK-293A cells transduced with rAds encoding these proteins, while the degree of enhancement varied among the mutated versions.

To clarify the mechanism of the enhancement as well as the limitations of this study, it would be necessary to see (i) whether similar enhancement can be observed in HPV E6/E7-immortalized epithelial cells that show higher dependence on Pentamer for GPCMV entry^18^ and in cell types other than epithelial cells, (ii) whether the enhancement is associated with immortalization of epithelial cells with SV40 T antigen that was reported to inhibit HCMV infection at multiple points in fibroblasts^37^ and result in poor GPCMV infection^18^, (iii) the target molecules for GP131/GP133-mediated enhancement, and (iv) whether this enhancement reflects any process that occurs during the Pentamer-dependent signaling pathways. It is common in the field of virology that efficiency of infection in one cell type is different from that in another cell type. Although such differences are usually explained by differences in the viral entry or traffic process, this study suggests the presence of an additional mechanism that controls the efficiency of infection. Nevertheless, identification of the cellular targets remains critical for a better understanding this novel mechanism.

In conclusion, we identified an unexpected phenomenon in which endogenous co-expression of two components of Pentamer enhanced infection, probably through increase of IE-1/2 expression from the viral genome delivered to the nuclei. Although our findings represent an artificial enhancement of infection, we believe further studies to clarify the details may open a path to understanding the mechanisms of viral tropisms and determinants that control the efficiency of viral infection of cells.

## Materials and Methods

### Cells and viruses

GPCMVd9K (WT) expressing GFP and its mutants GP129Stop (Δ129) and GP131Stop (Δ131), which contain an early stop-codon mutation in the GP129 and GP131 genes, respectively, were reported previously^17^. Production and titration of their viral stocks were performed in guinea pig fibroblast GPL cells (CCL158, ATCC). Although the infectivity depended on cell types, we used the GPL-based titers to calculate MOI. Guinea pig epithelial cell line GPE-7 was established previously by immortalization of primary renal cells with SV40 T antigen followed by cloning of cytokeratin-positive cells^19^. Infection of most GPE-7 cells with GPCMV required 100-fold amount of the viruses for infection of GPL cells. HEK-293A (Invitrogen) and the guinea pig epithelial cell line GPC-16 (CCL-242, ATCC) were purchased commercially.

The genes encoding the GPCMV Pentamer components, gH, gL, GP129, GP131, GP133, and GP131 with a charged amino acid-to-alanine alteration were cloned into pENTR3C (Invitrogen) respectively. rAds encoding each Pentamer component were constructed by the Gateway cloning from the pENTR3C constructs into pAd/CMV/V5-DEST (Invitrogen) followed by transfection of PacI-digested plasmids into HEK-293A cells^19^. The rAd titers were measured in HEK-293A, and β-galactosidase staining of the cells transduced with rAd-LacZ indicated that infection at an MOI of 20 was required to transduce all GPE-7 cells.

### Immunological detection of GPCMV and its products

Polyclonal anti-GPCMV IE1/2 and anti-gB antibodies^13^ and monoclonal antibody against a GPCMV early antigen g-1^21^ were used for the immunological detection of GPCMV products. Immunostaining of infected cells in the wells of culture plates was performed as follows. Infected cells were fixed with 3.7% formalin, treated with 0.05% TritonX-100 in phosphate-buffered saline (PBS), rinsed with PBS, and incubated with a dilution of g-1, anti-GPCMV IE1/2 or anti-gB antibodies. After washing three times with PBS, the cells were incubated with horseradish peroxidase-conjugated anti-rabbit or mouse immunoglobulin G antibody (Histofine Simple Stain MAXPO, Nichirei, Japan) at 37 °C for 1 hr. Positive cells were visualized with 3,3′-diaminobenzidine (DAB substrate, Roche).

### Preparation and detection of BrdU-labeled GPCMV

One day after the infection of GPL cells with viral stocks, the infected cells were cultured in medium containing 10 µM BrdU (Sigma Aldrich). Cell-free BrdU-labeled virus stocks were prepared by the sucrose step gradient centrifugation of the culture supernatants of the infected cells. Infectious titers were measured in GPL cells. To detect BrdU-labeled GPCMV, the cells were grown and infected in 8-well LabTek chamber slides (glass) (ThermoFisher), and then the slides were treated with 4 M HCl for 10 min to denaturate DNA, rinsed with PBS, and reacted with anti-BrdU monoclonal antibody (3D4, Becton Dickinson) followed by FITC-conjugated rabbit anti-mouse IgG polyclonal antibody (DAKO). Evans blue was used for counter staining. After the reactions, BrdU signals on the plasma membrane were observed under a confocal microscope. Images of 20 fields (0.17 μm^2^/field) for each set of conditions were captured, and the virus signals were counted.

### Preparation and detection of DiO-labeled GPCMV

DiO (Vybrant cell-labeling solution, Molecular Probe) was added to a GPCMV WT stock (4.1 × 10^7^ PFUs in 180 μl of PBS) that was partially purified by sucrose gradient centrifugation^15^, at a final concentration 2 μM, and the mixture was incubated for 1 hr at room temperature. The mixture was used as the DiO-labeled GPCMV stock. GPE-7 cells in 8-well chamber slides (Nunc) were incubated with the DiO-labeled GPCMV at an MOI of 22.5 for 1 hr at 4 °C, and then the inoculum was replaced with warmed culture medium. The cells were incubated for the indicated duration at 37 °C, fixed with 3.7% formalin, stained with DAPI (NucBlue Fixed Cell Stain Ready Probes reagent, Life Technologies) for detection of nuclei and with 300-fold diluted acti-stain 555 fluorescent phalloidin (Cytoskeleton, Inc.) for detection of the polymerized form of F-actin, and observed under a confocal microscope. Images of 10 fields (0.17μm^2^/field) for each set of conditions were captured, and the virus signals were counted.

### FITC-labeled materials and inhibitors for endocytosis pathways

FITC-labeled-CTB, and -dextran (MW 70-kDa) were purchased from Sigma and used at final concentrations 10 μg/ml and 3 mg/ml, respectively. Inhibitors of endocytotic pathways, genistein (Wako, Japan), dynasore (Cayman Chemical, MI, US), and latrunculin (Wako), were used at final concentrations, 200, 50, and 2.5 μM, respectively.

### Determination of viral loads

Preparation of the nuclei of the GPCMV-infected cells and purification of DNA from the nuclei were performed as described previously for VZV-infected cells^38^. Quantitative PCR was performed as described previously^13^.

## Data Availability

The datasets generated and/or analyzed during the current study are available from the corresponding author on reasonable request.
